# Renovation of Sick and Wounded and Restoration to Forward Area

**Published:** 1918-12

**Authors:** 


### RESTORATION OF THE SICK AND WOUNDED TO THE LINE

**Renovation of Sick and Wounded and their Restoration to the Forward Area by Methods usually Followed in Convalescent Depots.** Colonel Dalrymple, B. A., in France.

The speaker said in part :

It is necessary at the outset to understand thoroughly what is meant by a *Convalescent Depot.* The word " convalescent " has always been to my mind somewhat of a misnomer, and yet, as we are a Medical Unit and its inhabitants not quite normally fit men, it could not very well be called a training depot or a rest camp. People who have not seen a Convalescent Depot may have visions, when the word convalescence is used, of people reclining in comfortable chairs or hammocks, wearing silk dressing jackets, being served with stimulating beverages, and doing nothing else except laze through the idle day.

The Convalescent Camp, Gentlemen, as we understand it, is quite a different thing. It is a depot pitched conveniently adjacent to hospitals, generally having a capacity for either 2,000 or 5,000 men, and into which the hospitals send cases no longer requiring hospital treatment and the attentions of a skilled nurse, where the man can take his ordinary diet, can wash and clothe himself and walk about, in fact where the man suffers only from a feeling of slackness due to a slight wound or other disability, and requires a little toning mentally and physically to fit him for the arduous duties of a front line fighting man. We are, Gentlemen, the transition stage between Hospital and the Base Depots, from which re-inforcements are drawn. Not only are such cases admitted as I have described, but in times of pressure, with large convoys of sick and wounded coming down the line, we take in cases of lightly wounded requiring dressings which can be done by orderlies under the supervision of a Medical Officer. In the Depot under my command we have had as many as 1,500 daily dressings of this nature, and excellent progress has been made. We also take men suffering from minor injuries, sprains and bruises, myalgia, mild pyrexias, in fact all men who do not require skilled nursing, and where it is not necessary that they should occupy a bed more urgently required by really sick or wounded men. From an experience of commanding a Convalescent Depot for 2 1/2 years, I often think that hospitals might make a much greater use of us, and many cases coming down the line need never be admitted but sent direct to us. There is one very important point to remember, a point which applies to probably 75 per cent. of the men admitted, and which considerably alters your treatment of them. That is that these men come in having a grievance, not a justifiable one as it appears to us, but to them distinctly a grievance, and that grievance is that they were not sent to England, that their wound was not severe enough or their illness not marked enough to warrant the O. C. of the hospital from which they came to send them home. They know that they have come to the " Con. Camp " as they call it, to be made fit to return to the front for the resumption of business. The majority of them, good-hearted, sporting fellows, will blind their fate and curse their luck for a short time, and then throw themselves into the life of the Depot and get fit; a small minority have to be encouraged to do so. It is this condition of mentality, a mixture of disappointment, depression, and vituperation, which all officers of Convalescent Depots should thoroughly grasp and understand, and make it their chief aim to remove by all the methods in their power. We have also to remember that this man is away from his Regiment, away from his friends, mixed up with men of Units he has probably only heard of and never seen; they are all strange to each other. If he had been wounded badly he would have gone home to friends; had he been sent back from the Field Ambulance to his Regiment, he would have gone back to his " pals "; but here to a " Con. Camp "! " Why he knows no-one! " You will pardon me, Gentlemen, for dwelling upon this point, but the great secret of succeeding with these men is to know your material well. No builder, artisan or engineer, nor any other useful man ever commences work without first examining his material, therefore my advice is — study and understand your *man* on his admission to your depot.

The best conducted Convalescent Depots are run on the Brigade or Battalion system with its Companies which an officer commands, and under him a Company Sergeant-Major with a Sergeant to each hut, say of 20 men, or if Sergeants are scarce Corporals will do. The Sergeant-Majors are part of the permanent staff of the Depot; the Sergeants or Corporals may be convalescents.

The speaker then gave a detailed description of an inspection of the formation, laying special emphasis on the need of careful examination for pediculi and their eggs. He continued :

There is a right and a wrong way of taking this parade, and a great deal will depend upon your output of properly refitted troops, physically and mentally equipped as a first class fighting man should be, and upon your correct attitude at this preliminary inspection. You may walk down the line with a face like an owl, hearing the man's reply to your question of " What is the matter with you? ", giving the treatment to your orderly, telling the Sergeant to get that man's hair cut, and taking absolutely not a bit of interest in the man standing looking at you. This is wrong, Gentlemen, it is criminal. For God's sake be human; take some interest in these fellows; the majority of them are men who have been wounded some time or other, who have been through trials which, in pre-war days, we never dreamt human flesh could stand. Ask him about his country, " Which part of Canada or South Africa or America do you come from? " " What do you do there? " Or to a home-born man, " Where do you come from; what do you work at? " or " What is your trade in civil life? " " Do those boots fit you. " " They seem too tight? " " You will lose that button very soon; better go to the tailor's shop and get it fixed! ", and so on. Take an interest in each man and get them to smile.

Your next duty is to see these men sorted out into trades. As a Convalescent Depot has a very limited staff of men, it is necessary to use some convalescents for an hour or so each day in depot work, and as far as possible men should be given jobs which they have been used to in civilian life. I also find that for very tired men it is a real rest and convalescence is hastened by employing them at their own jobs. The men who will, eventually, be restored to their regiments in the front line, are occupied by physical training parades, recreational exercises and games, which I shall touch upon later. The labor type of man, who could never become a first class fighting man, you use for fatigues and work for which he is normally suited. With regard to the age stated, this of course all depends upon how you find each man. You occasionally find men, particularly those who belonged to the regular forces prior to the war, and who have been trained, better at 40 than the men who have never been trained as soldiers, and who probably have earned a precarious living in some large town, are at 25. This is where your experience and knowledge of mankind helps you.

There are certain essentials to be observed in your treatment of convalescents in order to ensure success. The first thing is to treat them as *soldiers* and not *patients*. Let that be distinctly understood; let him understand that he is no longer in hospital, but is back in his uniform, of which you must encourage him to be proud; and discipline of not too irksome a nature must be enforced. There is a tendency for the soldier to be rather spoiled in hospital, especially if he is an obliging or decent sort of fellow, and if his stay there has been a long one, particularly also if he has been employed in some capacity in the wards, kitchens or stores of a hospital. It is your duty to remove this semi-invalid attitude, restore to him his self-respect and appreciation, and then restore him to his Regiment. I should recommend that you and your officers give your undivided and wholehearted attention to the following departments of your Depot. I have placed them under headings starting with the one I consider of greatest importance; the first department to which particular attention should be given is food.

1. Food.
2. Quarters.
3. Cleanliness (change of clothes, baths).
4. Amusements and games.
5. Physical training, dancing, recreational exercises.
6. Shooting.

On the subject of amusements the speaker said :

It is difficult to say amusements come third in importance because physical training and recreational exercises are also amusements, in my opinion, or should be if your arrangements are perfect. There is nothing so dog-tiring and thought-paralyzing as what is known as drill, just ordinary barrack square drill, particularly to the war-tired soldier man. It doesn't make him think — he does it automatically; he is not interested; it tires him and he wishes it were over. Physical training has come to take its place, so far as Convalescent Depots are concerned. It embodies what are known as recreational exercises, dancing to the music of the band, games, sports and all manner of diversions. The instructors create a desire and spirit of emulation among the men by having inter-company football, and cricket matches, basket ball, boxing contests, and so on. I saw it the wrong way, but after I had seen a few hundred men do some simple steps to the band, in the meanwhile laughing and happy, forgetting their old troubles, I approved of it. Don't force men to play games, encourage them, get hold of the right instructors, and above all supervise yourself.

There are other indoor amusements, of course, which are also valuable in restoring a man's thoughts to a cheerful and restful outlook — concerts, theatricals and music. We run a Drum and Fife Band, String Orchestra of 20 performers and bag pipes for marching small parties. There should be a concert or theatricals every night in your depot, a boxing contest, a skittle match, a lecture, or some other attraction. Try to make your camp so attractive that the men won't want to leave it for the adjacent town or neighborhood in which to pass their spare time. A miniature shooting range has been established in No. 3 Depot, and besides being a source of great attraction it also helps to improve their marksmanship. I believe also in making a man's surroundings pleasant; for instance, — we go in for lawns and flower gardens and insist upon neatness and cleanliness around their huts. We also encourage them to make gardens for flowers, and prizes are given for the neatest and best-kept huts, including exterior and interior.

The daily amount of work demanded from a convalescent should not be too great or too irksome. We have reveille at 5-30 a.m., and at 7 a.m. the whole Depot parade with the band to see that all men are washed and cleaned up, and also to get half an hour in the fresh air before breakfast, which is at 7-45 a.m. At 9 a.m. the whole Depot again parade and men are then told off, some for physical training, some to fatigues, and others who require dressing for slight wounds, and so on. Physical and recreational training goes on until 11-30 a.m., when the men fall out and retire to the various auxiliary institutes we have in the Depot, such as the Y. M. C. A., Salvation Army, and Red Cross Hut, where they may play billiards, cards and other indoor games, read, or write letters. Dinner at 12-45 p.m., fall in again at 2 p.m. for dancing to the band, voluntary games, etc. After 3 p.m. the men are free to follow their own desires. They nearly all play games, generally football, and there are often three matches going on at once.

It is just as well to make the boundaries of your camp as liberal as possible and so obviate the incentive to break bounds. I do not agree with very limited bounds or boundaries marked by barbed wire giving the place a prison-like appearance. It develops a sense of restriction in the bold spirit, with subsequent probable breaches of discipline.

I forgot to mention earlier that it is necessary to obtain and issue on a liberal scale in the reading rooms of your institutes quantities of papers, magazines and periodicals, and also to establish a library. There are numerous societies in England only too ready to supply reading matter for this purpose. It is also as well to have a trained chiropodist in your Depot as well as a masseur.

The average stay in the Depot is from 14 to 21 days; some stay longer. It entirely depends upon the man's physical condition. Men are paraded on the seventh day after admission to see if they are fit for discharge, and, if not then, every following seventh day, unless their company officers, who have them constantly under supervision, consider they are fit to go out sooner. Men leaving the Depot are either marked " A " if fit, or " M. B.", which means they proceed to Medical Board Base Depots to be classified.

As I have pointed out before, it is necessary to have good discipline, but this should not be of too irksome or irritating a character. When a man is on parade or on duty, by all means have perfect discipline, but at other times let the restraint be sufficient to ensure that his conduct is that of a soldier.

During the passage of over 65,000 men through the Depot I have the honor to control, crime has been at its minimum, and there have only been 4 trials by Courts Martial : 2 of these were for theft, and 2 for drunkenness.

